# Disrupted Co-activation of Interneurons and Hippocampal Network after Focal Kainate Lesion

**DOI:** 10.3389/fncir.2017.00087

**Published:** 2017-11-13

**Authors:** Lim-Anna Sieu, Emmanuel Eugène, Agnès Bonnot, Ivan Cohen

**Affiliations:** ^1^Institut de Biologie Paris Seine, UPMC/INSERM UMRS1130/CNRS UMR8246, Paris, France; ^2^Sorbonne Universités, Université Pierre et Marie Curie, Paris, France; ^3^Neuroscience Paris Seine (UMR-S 1130), Institut de Biologie Paris-Seine, INSERM, Paris, France; ^4^Institut du Fer à Moulin, UPMC/INSERM UMRS839, Paris, France

**Keywords:** epilepsy, hippocampus, interneuron

## Abstract

GABAergic interneurons are known to control activity balance in physiological conditions and to coordinate hippocampal networks during cognitive tasks. In temporal lobe epilepsy interneuron loss and consecutive network imbalance could favor pathological hypersynchronous epileptic discharges. We tested this hypothesis in mice by *in vivo* unilateral epileptogenic hippocampal kainate lesion followed by *in vitro* recording of extracellular potentials and patch-clamp from GFP-expressing interneurons in CA3, in an optimized recording chamber. Slices from lesioned mice displayed, in addition to control synchronous events, larger epileptiform discharges. Despite some ipsi/contralateral and layer variation, interneuron density tended to decrease, average soma size to increase. Their membrane resistance decreased, capacitance increased and contralateral interneuron required higher current intensity to fire action potentials. Examination of synchronous discharges of control and larger amplitudes, revealed that interneurons were biased to fire predominantly with the largest population discharges. Altogether, these observations suggest that the overall effect of reactive cell loss, hypertrophy and reduced contralateral excitability corresponds to interneuron activity tuning to fire with larger population discharges. Such cellular and network mechanisms may contribute to a runaway path toward epilepsy.

## Introduction

GABAergic interneurons are crucial in the regulation of principal cell networks, through inhibitory control loops in different type of physiological activities (Buzsáki, 2001; Somogyi and Klausberger, 2005; Kullmann, 2011). They modulate synchronized physiological network activities like sharp-waves (Buzsáki et al., 1992; Traub et al., 2000; Maier et al., 2003; Ho et al., 2009; Rácz et al., 2009; Ellender et al., 2010) that are generated through recurrent synapses of CA3 principal cells (Buzsáki, 1986), and coordinate oscillatory synchronized physiological activity by participating in different phases of network oscillations (Whittington et al., 1995; Traub et al., 1996; Klausberger et al., 2003; Gulyás et al., 2010).

In temporal lobe epilepsy (TLE), a profound neuronal reorganization affects the balance between excitatory principal cells and interneurons. In the hippocampus, principal cells are lost in area CA1–CA3, while the dentate gyrus exhibits axonal sprouting and neurogenesis (Tauck and Nadler, 1985; Cronin and Dudek, 1988; Sutula et al., 1988; Goodman and Sloviter, 1992; Isokawa et al., 1993; Wenzel et al., 2000). Similarly, interneurons present specific patterns of cell loss, preferably for somatostatin containing neurons (Sloviter, 1987; Buckmaster and Dudek, 1997; Buckmaster and Jongen-Rêlo, 1999; Cossart et al., 2001; Halabisky et al., 2010; Marx et al., 2013) and widespread synaptic reorganization, including in parvalbumin containing neurons (Nakajima et al., 1991; Wittner et al., 2001; Dinocourt et al., 2003). In the rodent *in vivo* model of unilateral intra-hippocampal kainate (KA) injection, which reproduces key features of TLE (Bouilleret et al., 1999), the selective glutamate agonist triggers an acute wave of hyperactivity which is only slowed down by inhibitory transmission (Le Duigou et al., 2005). Over time, ipsilateral hippocampus areas distal from the injection site show spontaneous interictal-like population activity, that remain present when tissue slices are kept in an *in vitro* interface chamber (Le Duigou et al., 2008). This activity pattern is similar to that observed in tissue resected from TLE patients in the subiculum, although in the latter case GABA acts as a contributing factor rather than a brake to activity (Cohen et al., 2002). Thus, TLE tissue shows complex remodeling of inhibitory circuits, which may contribute to alter the powerful patterning role they have under physiological conditions.

In this study, we established an experimental protocol to explore functional remodeling of inhibitory circuits in epileptogenesis, based on the kainate mouse model of TLE. We first induced epileptogenesis by a unilateral kainate injection into the dorsal hippocampus *in vivo.* Two weeks later, at a time corresponding to the emergence of spontaneous seizures in this *in vivo* model (Riban et al., 2002), *in vitro* recordings were performed in hippocampal slices. We used a modified submersion-type recording chamber (Hájos et al., 2009) to preserve spontaneous neuronal population discharges *in vitro* (Hájos and Mody, 2009) and we combined patch-clamp recordings from interneurons with extracellular recordings that monitor synchrony of neuronal populations (Cohen et al., 2006). Mice expressing a green fluorescent protein (eGFP) under control of a glutamic acid decarboxylase (GAD67) promoter were used in order to selectively patch interneurons.

We found spontaneous extracellular potentials (SEP) both in control and KA treated mice, presumably corresponding to network discharges (Cohen et al., 2006). While amplitude distribution was similar between control and KA-treated groups for SEPs of small amplitude, KA treated mice displayed larger amplitude events, with a cutoff around 400 μV. They had fewer interneurons, which showed a significant increase in soma size. Interneurons had decreased resistance and increased capacitance and required higher current intensity to fire action potentials. Unlike control tissue, interneurons only rarely discharged with small amplitude SEP, in this *in vitro* preparation, while they were recruited by large amplitude SEP. Altogether, these observations suggest that the overall effect of reactive cell loss, hypertrophy and reduced contralateral excitability corresponds to interneuron activity tuning to fire with larger population discharges.

## Materials and Methods

### Animals

All animals received humane care in compliance with the European Communities Council Directive of 2010 (2010/63/EU), and the study was approved by the institutional and regional committees for animal care. Experiments were performed on 25 adult (4–10 weeks old) male mice from the GAD67-GFP (delta neo) line (Tamamaki et al., 2003). The mice expressed eGFP protein under control of the GAD67 promoter, allowing fluorescent labeling of GABAergic interneurons in live tissue. Male animals were used similarly to earlier studies in order to restrain variability (Bouilleret et al., 1999) and recent analysis has confirmed the rationale for this selection due to differences related to sex in this model (Twele et al., 2016).

### Kainic Acid Injection

**FIGURE 1:**
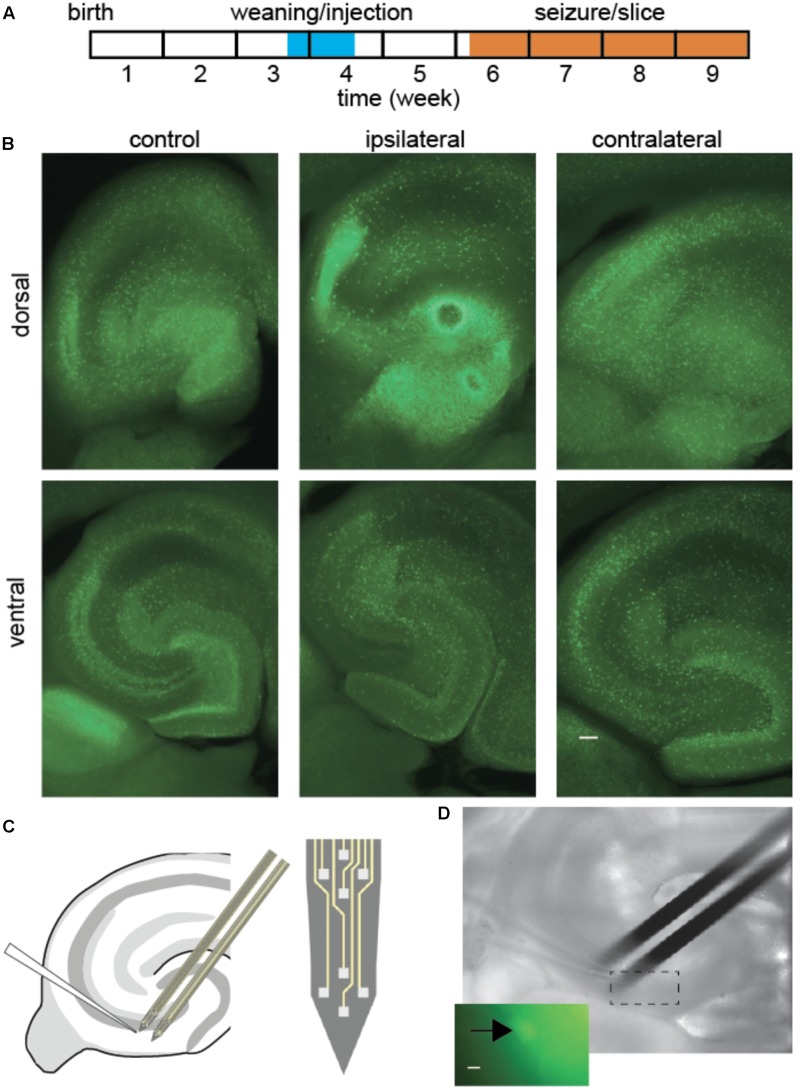
Interneuron and extracellular potential recording after epileptogenic lesion. **(A)** Experiment timeline. **(B)** Fluorescence ipsilateral and contralateral to focal KA lesion, 2 weeks post-injection. Overall fluorescence was most intense around the lesion (dorsal ipsilateral), although in a diffuse pattern. Cell bodies remained visible throughout most tissue, while anatomical layer boundaries were blurred in KA-treated animals, most prominently on the ipsilateral side. Scale bar 100 μm. **(C,D)** Schematic and snapshot of patch-clamp recording from fluorescent interneuron in CA3 and nearby population local field potentials with extracellular silicon probes. Scale bar 5 μm.

Experiment design required a delay between kainic acid lesion and *in vitro* tissue recording (**Figure 1A**). Mice 3–4 weeks old, 3–5 days after weaning, were anesthetized by isoflurane inhalation (1–1.5%) and placed in a stereotaxic frame. A droplet of 50 nl of KA solution was injected in the right dorsal hippocampus (from the bregma: anteroposterior -1.8 mm, mediolateral -1.8 mm, dorsoventral -1.8 mm) through a fine needle of outer diameter 0.1 mm (Hamilton, NV, United States), connected to a 10 μl syringe, mounted on an electric pusher (WPI, Sarasota, FL, United States). The KA solution contained: KA (20 mM, Sigma–Aldrich), NaCl (0,9%) and Ruby Red dye (*M* = 3000, 10 mg/ml, Invitrogen), so that the site of injection could be identified. The injection procedure required 30–40 min of isoflurane anesthesia. In this model, the injection site is central between Dentate Gyrus, CA3 and CA1, as kainate diffuses from this point to lesion the dorsal hippocampus. As expected the lesion was observed in the dorsal tissue ipsilateral to the injection (**Figure 1B**), and the fluorescence distribution was altered by the lesion. Since mice in the control group did not receive saline injection, it cannot be excluded that some of the changes observed could be related to the injection procedure rather than to the epileptogenic effect of kainate. In those animals, GAD67-GFP interneurons spread across all areas and layers of the hippocampus. In KA-treated animals the injection site was sclerotic, and the anatomical layers became less visible in the dorsal hippocampus, while in the ventral hippocampus the tissue was less affected.

### Slice Preparation

Slices were prepared 2–3 weeks post-injection, or at the corresponding age of 5–7 weeks in untreated animals. In the kainate lesion model, spontaneous seizures appear 2 weeks after the lesion. We did not check whether each animal presented seizure at the time of tissue preparation, because seizure time can be as low as a few minutes per 24 h. However, we performed the routine check for this model to observe tissue lesion at the injection site, as slices were cut. Animals were anesthetized by brief inhalation of halothane and were then decapitated and their brain removed. A tissue bloc containing left and right hippocampi was cut in horizontal slices of 350 μm thickness with a vibratome (Microm HM 650V, Walldorf, Germany) in a cold solution (2–4°C) containing (mM): choline chloride (110), KCl (2,5), NaH2PO4 (1,25), NaHCO3- (25), D-Glucose (7), CaCL2 (0,5) and MgCl2 (7). Slices were then transferred to a storage interface-type chamber (35°C), where they were placed in an artificial cerebro-spinal fluid (ACSF) solution containing (mM): NaCl (125), KCl (2,5), NaHCO3- (26), D-Glucose (10), MgCl2 (2) and CaCl2 (2), while their upper surface was exposed to a humidified O2/CO2 95/5 atmosphere (Maier et al., 2009). Cutting and ACSF solutions were oxygenated with O2/CO2 95/5 gas mixture (pH 7,4). One slice was transferred to the submerged recording chamber and immobilized with a nylon mesh. Continuous perfusion was performed with oxygenated ACSF, 10 ml/min at 32°C. Perfusion flow reached both the lower and the upper sides of the slice (Hájos et al., 2009). This recording condition preserved spontaneous activity. The chamber was placed on an Axioscope 2 FS microscopy (Zeiss) platform, equipped with a 40× DIC objective. Green fluorescent GAD67 interneurons were visualized using selective filters (Ex: 485/20 nm, Em: 530/25 nm).

### Extracellular Recordings

Neuronal population activity was recorded with a 16 channels silicon extracellular electrode (NeuroNexus, Ann Arbor, MI, United States), mounted on a micromanipulator. The electrode was composed of two branches spaced by 150 μm, each branch comprising two tetrodes (**Figure 1C**). Each electrode channel could detect spontaneous local field potentials, including extracellular multiunit action potentials (Cohen and Miles, 2000) and extracellular synaptic potentials (Bazelot et al., 2010). The silicon electrode was placed in CA3 with one tetrode in *Stratum Oriens*, two tetrodes in *Stratum Pyramidale* and one tetrode in *Stratum Radiatum*. Signals were amplified 1000 times and filtered between 1 Hz and 20 KHz, by a 16 channels amplifier with high input impedance (Xcell, Dipsi, Châtillon). Noise amplitude ranged from 5 to 10 μV, while action potentials, observed in *Stratum Pyramidale*, ranged from 40 to 200 μV.

### Intracellular Recordings

Interneurons were studied with the patch-clamp technique in the whole-cell configuration. Borosilicate pipettes were filled with a solution containing (mM): *K*-Gluconate (130), KCl 5), HEPES (10), EGTA (10), MgCl2 (2), Mg-ATP (4), Tris-GTP (0,4), Na2-phosphocreatinin (10), and biocytin (0,1%). The pipette resistance ranged from 2 to 6 MΩ in ACSF. This solution generated a non-corrected liquid junction potential of -15 mV. All chemicals were purchased from Sigma–Aldrich. An Axopatch 200A amplifier (Molecular Devices) was used in current-clamp mode to establish whole-cell configuration. Data were digitalized by a 16-bit analogic-digital conversion interface with 16 channels (Digidata 1322A, Molecular Devices), and displayed with the accompanying software Axoscope.

### Signal Analysis

Interneuron intrinsic properties were derived from current-clamp recording. Resting potential was obtained by averaging membrane potential for 10 s when no current was injected. Membrane resistance and capacitance were measured from hyperpolarization steps of 50 pA. We computed the average of 10 traces without synaptic events. An exponential curve was adjusted to obtain values for time constant, resistance and capacitance. Then, membrane potential was adjusted to -65 mV and a series of current steps lasting 1000 ms were applied. We counted the number of elicited action potentials generated and identified the threshold values as the intersection of the linear fit of the frequency-injected current (F-I) curve with the axis of current intensities. Input-output gain was computed as the slope of the linear fit.

Extracellular signals recorded from each electrode were filtered to isolate local field potentials (SEP, 5–300 Hz) characteristic of synchronous activities. They were automatically detected by an algorithm previously presented in (Cohen and Miles, 2000) that gave a proportion of false positives and false negatives that did not exceed 3%. We compared the characteristics of SEP measured in *Stratum pyramidale*, using a single electrode site from each multichannel recording. SEP duration was estimated as the time of the rising phase of the spike, which is less sensitive to noise than the full spike-wave duration.

We distinguished SEP of amplitude less than 400 μV that had similar distribution in control and KA-treated tissue and those of larger amplitude that were at least an order of magnitude more abundant in epileptic tissue. Small SEP events correspond to synchronization of only a fraction of neurons, while large SEP events correspond to massive synchronization (Cohen et al., 2006). The detection of SEP signals from recording sites located 200 μm apart implied that both small and large SEP synchronous activities were taking place across spatially extended neuronal populations. In order to compare the SEPs that we observed with reported spectral signatures of sharp waves (Foffani et al., 2007), we computed the spectrum of SEP of small and large amplitude (Supplementary Figure 1). Spectra were computed by averaging STFTs of individual SEPs as in Foffani et al. (2007), with additional high-pass filtering at 50 Hz (Bessel 4th order in forward and reverse time directions) to attenuate the dominant low-frequency component and better reveal ripple or potential fast-ripple components.

Signal analysis was performed with routines implemented using Labview (National Instruments, Austin, TX, United States). Since the kainate lesion was unilateral, we distinguished three groups: control tissue, tissue ipsilateral and ventral relative to the lesion, and contralateral tissue. Combined patch-clamp and extracellular recordings analysis was performed on a total of 25 cells from 22 slices, 15 animals (8 control and 10 with kainate lesion). Numbers per group are: Control (10 cells from 9 slices, 8 animals), Contralateral (6 cells from 6 slices, 5 animals) and Ipsilateral (9 cells from 5 slices, 5 animals). One animal was prepared per day, yielding recordings from 1 or 2 slices. For kainate lesioned animals, when two slices were recorded the same day, they could be either from same or opposite sides, which was chosen before preparing the tissue and aimed at ensuring groups had similar sizes. Recording lengths were of comparable size, ranging from 30 to 45 min. For intracellular properties in control animals, we pooled another 12 patch-clamp recordings from another 10 slices, 9 animals.

### Morphology

During intracellular recordings, the cell was filled with biocytin (0,1%) by diffusion from the pipette. After the recordings, each slice was fixed in a 4% Paraformaldehyde solution, dissolved in a 0,12 M phosphate Buffer (PBS) solution, and left under continuous agitation at 4°C during 24 h. Slices were then transferred to a 0,12 M PBS solution, sucrose (30%) at 4°C for cryoprotection and each slice was left for at least a night under agitation. Freezing-unfreezing (x3) on dry ice was then used to permeabilize the membranes, followed by three washes (PBS 0,12 M at ambient temperature). Saturation and permeabilization were performed with PBS 0,12 M, milk (0.2%), Triton 100X (0.3%), for 3 h at ambient temperature. Slices were then incubated overnight with 1/500 Streptadivin-Cy3 (Invitrogen) and 1/1000 DAPI in PBS 0,12 M, milk (0.2%), Triton 100X (0.1%) at 4°C. The Streptavidin revealed biocytin (avidin-biotin complex) whilst DAPI revealed cellular nuclei. After three washes with PBS 0,12 M, the slices were mounted in a Prolong Gold Antifade medium (Molecular probes, Invitrogen) that preserved fluorescence. Cy3, GFP, and DAPI were visualized with a fluorescent microscope equipped for structured light imaging (Optigrid system, Qioptiq). Acquisition was made with 4× or 20× lens with a high-resolution camera (Qimaging Retiga EXi 1392 pixels × 1042 pixels). The images were scanned and visualized using Volocity software (Improvision, Perkin Elmer).

### Cell Counting

Cell counts and soma size estimation were performed on cumulative stacks of 350 μm thickness, using the GFP fluorescence in this mouse line. CA3 sectors were divided according to *Cornu Ammonis* layers. We pooled *Lacunosum Moleculare* and *Radiatum* as the exact border between the two was not always sharp. Counts were divided by the volume examined to obtain a cellular density, in order to compensate for possible hippocampal KA-induced shrinkage (Wolf et al., 2002).

### Statistics

Statistical analysis was performed with Labview and R language and environment for statistical computing (R Core Team, 2017).

Spontaneous extracellular potential features are normalized for recording length, so that number of event expected for a given amount of time, for a range of the variable, corresponds to the area under the curve multiplied by the time measured in seconds. For distributions relative to firing we used Kolmogorov–Smirnov two-sample test and Bonferroni correction for multiple testing.

Tissue and cellular properties, may have non-Normal distributions, which require non-parametric test. We used Anderson–Darling two-sample test, which offers good power even for limited sample size, and Benjamini–Hochberg procedure for multiple testing.

## Results

The CA3 network was studied after a delay that corresponded to the early phase of epileptogenesis, around 2 weeks after a focal KA lesion in the dorsal hippocampus of the right hemisphere (**Figure 1**). We compared slices ipsi- or contralateral to the lesion with control tissue.

### Spontaneous Population Activity

**FIGURE 2:**
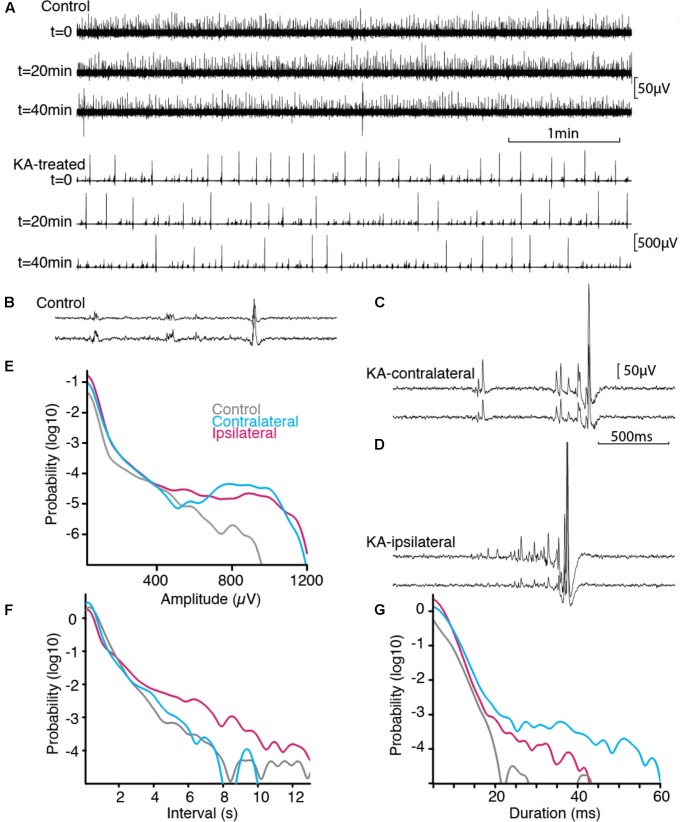
Spontaneous activity *in vitro*. **(A)** Stable spontaneous activity was observed in our immersed slice preparation in physiological saline, both in control and epileptic tissue, example shown from ipsilateral. Sample extracellular traces are shown at 20 min interval. B-D, Activity pattern differed between control **(B)**, contralateral **(C)**, ipsilateral side **(D)**. Scale bars common to **(B–D)**. Paired extracellular recording show synchronous SEPs across distance of 180 microns in *Stratum Pyramidale*. **(E–G)** Normalized distributions of SEP features across all recordings (*n*_CTRL_ = 115507 from 8 animals, *n*_CONTRA_ = 107141 from 5 animals, *n*_IPSI_ = 224497 from 5 animals). Epileptogenic tissue displayed more events of larger amplitudes and of longer duration, separated by smaller intervals. Prolonged silent periods were more prominent ipsilateral to the lesion.

Spontaneous neuronal activity was assessed in acute slice preparation by recording spontaneous extracellular SEPs, in control tissue, and ipsilateral or contralateral side of KA lesion (**Figure 2** and **Table 1**). Slices exhibited stable spontaneous activity for prolonged recording sessions (>1 h). Both control and KA slices exhibited spontaneous SEP discharges (**Figure 2A**). These discharges spanning a wide range of amplitudes were similar to those induced pharmacologically in acute experiment, for which amplitude is indicative of partial or extensive synchrony of neuronal populations (Cohen et al., 2006).

**Table 1:** Comparison of spontaneous discharge properties.

Test	A	B	Maximum distance	*p*
Amplitude	CTRL	CONTRA	0.021	<0.01
	CTRL	IPSI	0.025	<0.01
	CONTRA	IPSI	0.012	<0.01
Duration	CTRL	CONTRA	0.236	<0.01
	CTRL	IPSI	0.123	<0.01
	CONTRA	IPSI	0.113	<0.01
Interval	CTRL	CONTRA	0.139	<0.01
	CTRL	IPSI	0.106	<0.01
	CONTRA	IPSI	0.067	<0.01


*Kolmogorov–Smirnov test, *n*_*CTRL*_ = 73158 SEP, *n*_*CONTRA*_ = 68315, *n*_*IPSI*_ = 145864. Bonferroni-corrected *p*-values.*

Activity patterns were different in the three groups (**Figures 2B–D**). Control slices exhibited discharges of small amplitudes (**Figure 2B**). Epileptic slices further exhibited large amplitude discharges together with short episodes of ramp up activity (**Figures 2C,D**). We pooled the SEPs detected in our recordings to estimate the distribution of their characteristic shape and interval. Distributions were significantly different for amplitude, interval between discharges and duration (**Table 1**). While discharges below 400 μV had similar distribution pattern for all groups, the most prominent signature of epileptic tissue was an increased amount of discharges above 500–700 μV, rising to 10-fold, and more, above control tissue for larger amplitudes (**Figure 2E**). Thus, we distinguished small SEPs below 400 μV and larger SEPs that contribute to a distinct event distribution in epileptogenic transformation. Changes in interval distribution were more pronounced in tissue ipsilateral to the KA injection, with a ten-fold increase for intervals above 4 s (**Figure 2F**). Duration of SEP fell sharply in control tissue at around 20 ms, while both ipsi- and contralateral tissue distributed to larger values, with stronger difference on the contralateral side (**Figure 2G**). In order to compare SEPs with sharp waves reported earlier (Foffani et al., 2007), we computed the spectral signature of small and large SEPs (Supplementary Figure 1). In our protocole frequency spread of the low frequency component overlapped with potential ripple frequency (around 200 Hz), and would thus make further inference rely on modeling of the spectrum decomposition. A single peak around 300 Hz in the ipsilateral group fell below the range of fast-ripple (above 400 Hz).

### Interneuron Density and Size

**FIGURE 3:**
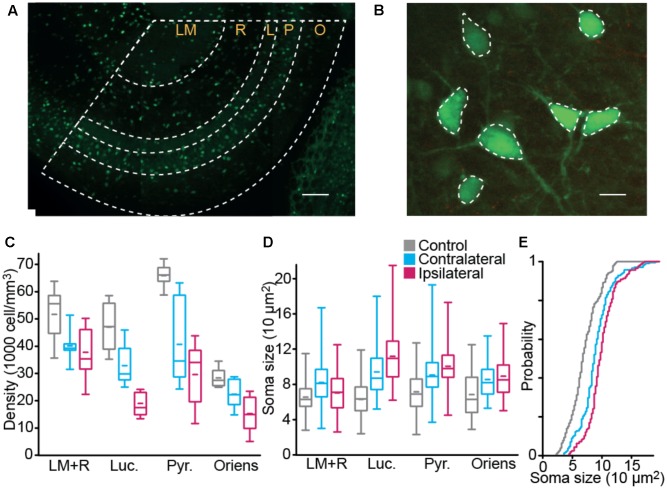
Interneuron density and size. Interneuron density **(A)** and body size **(B)** were estimated in ventral CA3 using GFP fluorescence in fixed tissue. Scale bars 100 and 10 μm. **(C,D)** Box plot distribution, over the three groups, of interneuron density **(C)** and soma size **(D)**. **(E)** Distribution of soma size in Stratum Pyramidale (*n*_CTRL_ = 9 animal, *n*_CONTRA_ = 6, *n*_IPSI_ = 9, “LM+R” *Stratum Lacunosum Moleculare and Radiatum*, “Luc.” *Stratum Lucidum*, “Pyr.” *Stratum Pyramidale*, “Oriens” *Stratum Oriens*).

Since interneurons exert a strong control on hippocampal network activity through inhibitory feedback, we compared interneurons without and with epileptogenic lesion, starting with their anatomical distribution (**Figures 3A,B**). To quantify these changes, we first counted the number of cells in stacks of images taken from our 350 μm slices (**Figure 3C**). Significant cell loss was found in the ipsilateral side, in all layers (**Table 2**). As expected, the damage was observed in the tissue closer to the lesion site, yet the contralateral side also presented changes, except in *Stratum Oriens*. Soma size (**Figures 3D,E** and **Table 3**) increased in all layers on both sides, relative to control. In *Lucidum*, both density and soma size were significantly different between ipsilateral and contralateral sides. Areas of greater cell loss coincided with stronger soma enlargement, suggesting that cellular hypertrophy took place to compensate for the functional deficit of inhibition induced by cell loss and synaptic reorganization.

**Table 2:** Comparison of interneuron density.

	A	B	nA	nB	T.AD	*p*	Rank	Valid
LM+Rad.	CTRL	CONTRA	9	6	2.770	0.02419	8	Yes
	CTRL	IPSI	9	9	3.634	0.01137	6	Yes
	CONTRA	IPSI	6	9	-0.4195	0.5745	12	No
Lucidum	CTRL	CONTRA	9	6	2.939	0.02081	7	Yes
	CTRL	IPSI	9	9	8.674	0.0001727	1	Yes
	CONTRA	IPSI	6	9	6.936	0.0006808	4	Yes
Pyramidale	CTRL	CONTRA	9	6	5.634	0.002067	5	Yes
	CTRL	IPSI	9	9	8.674	0.0001727	3	Yes
	CONTRA	IPSI	6	9	0.1484	0.3028	11	No
Oriens	CTRL	CONTRA	9	6	1.933	0.05145	9	No
	CTRL	IPSI	9	9	8.674	0.0001727	2	Yes
	CONTRA	IPSI	6	9	1.703	0.06361	10	No


*Anderson–Darling test, *p*-value, *p*-rank, Benjamini–Hochberg significance validity at alpha = 0.05. *n*_*CTRL*_ = 9 animal, *n*_*CONTRA*_ = 6, *n*_*IPSI*_ = 9. nA, nB number of cells for column A, B.*

**Table 3:** Comparison of interneuron soma size.

	A	B	nA	nB	T.AD	*p*
LM+Rad.	CTRL	CONTRA	630	294	81.12	<0.01
	CTRL	IPSI	630	396	22.04	<0.01
	CONTRA	IPSI	294	396	24.84	<0.01
Lucidum	CTRL	CONTRA	128	107	47.88	<0.01
	CTRL	IPSI	128	84	72.12	<0.01
	CONTRA	IPSI	107	84	11.26	<0.01
Pyramidale	CTRL	CONTRA	387	211	43.93	<0.01
	CTRL	IPSI	387	207	94.85	<0.01
	CONTRA	IPSI	211	207	12.76	<0.01
Oriens	CTRL	CONTRA	356	217	54.01	<0.01
	CTRL	IPSI	356	181	55.00	<0.01
	CONTRA	IPSI	217	181	1.362	1.05


*Anderson–Darling test, *p*-value, *p*-rank, Benjamini–Hochberg significance validity at alpha = 0.05. *n*_*CTRL*_ = 9 animal, *n*_*CONTRA*_ = 6, *n*_*IPSI*_ = 9. nA, nB number of cells for column A, B.*

### Interneuron Membrane Properties

**FIGURE 4:**
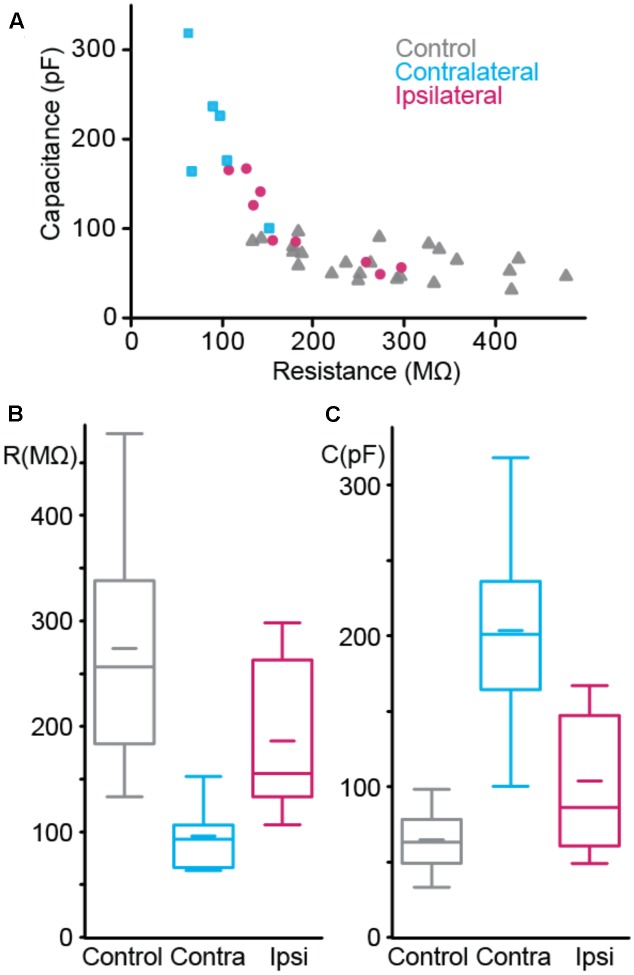
Passive membrane properties of interneurons. **(A)** Resistance vs. capacitance. **(B,C)** Boxplot of resistance **(B)** and capacitance **(C)**. *n*_CTRL_ = 22 interneurons, *n*_CONTRA_ = 6, *n*_IPSI_ = 9.

**FIGURE 5:**
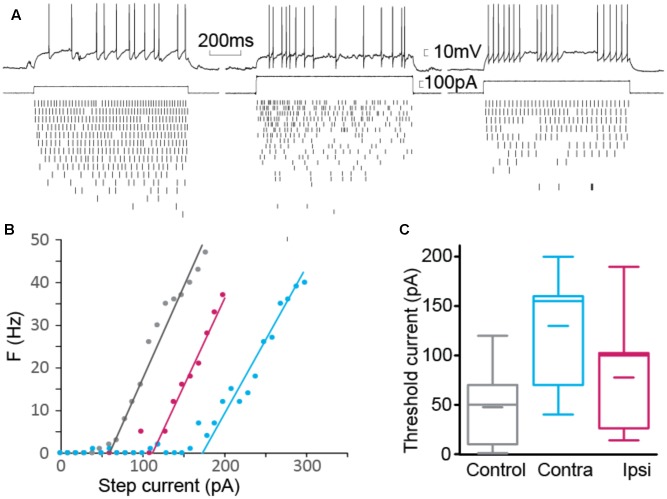
Firing threshold. **(A)** Example of elicited action potential firing in control (left), contralateral (center) and ipsilateral (right) interneuron. Current pulses of fixed duration (1 s) and varying amplitude were used. Action potentials were detected for each current step. **(B)** Number of action potentials as a function of step size. Threshold current was computed as the intersection of the linear fit with the current axis. **(C)** Summary for threshold current showing significantly larger current required to elicit action potentials in contralateral cells (*n*_CTRL_ = 22, *n*_CONTRA_ = 6, *n*_IPSI_ = 9).

We then asked if the changes in interneuron size could impact their intrinsic and firing properties. In order to limit the effect of interneuron diversity, we focused on cells with soma in *Stratum Pyramidale*, where cell density shows a strong decrease. This population includes basket cells that exert fast feedback inhibition on pyramidal cells (Miles et al., 1996). Intrinsic membrane properties were characterized using intracellular recordings of visually identified fluorescent cells (**Figures 4**, **5** and **Tables 4**, **5**).

**Table 4:** Interneuron passive properties and firing threshold.

	Control	Contralateral	Ipsilateral
Resting Vm (mV)	-60.1 ± 1.1	-60.7 ± 1.7	-59.6 ± 2.0
R(MOhm)	273 ± 21	96 ± 13	186 ± 24
C (pF)	64 ± 4	203 ± 30	104 ± 15
Threshold Ic (pA)	47 ± 8	120 ± 25	78 ± 19
Gain (Hz/nA)	377 ± 26	266 ± 37	293 ± 49


*Average ± SEM. *n*_*CTRL*_ = 22 interneurons, *n*_*CONTRA*_ = 6, *n*_*IPSI*_ = 9.*

**Table 5:** Comparison of interneuron passive properties and firing threshold.

	A	B	nA	nB	T.AD	*p*	Rank	Valid
Resting Vm	CTRL	CONTRA	22	6	-0.4059	0.5658	12	No
	CTRL	IPSI	22	9	1.280	0.0949	9	No
	CONTRA	IPSI	6	9	-0.8075	0.8617	8	No
R	CTRL	CONTRA	22	6	9.449	8.753e-5	2	Yes
	CTRL	IPSI	22	9	2.704	0.02566	7	Yes
	CONTRA	IPSI	6	9	4.695	0.004633	4	Yes
C	CTRL	CONTRA	22	6	10.64	3.139e-5	1	Yes
	CTRL	IPSI	22	9	2.763	0.02433	6	Yes
	CONTRA	IPSI	6	9	3.599	0.01171	5	Yes
Threshold	CTRL	CONTRA	22	6	5.510	0.002296	3	Yes
	CTRL	IPSI	22	9	0.8569	0.1439	11	No
	CONTRA	IPSI	6	9	0.9724	0.1283	10	No


*Anderson–Darling test, Bonferroni-corrected *p*-values. *n*_*CTRL*_ = 9 animal, *n*_*CONTRA*_ = 6, *n*_*IPSI*_ = 9. nA, nB number of cells for column A, B.*

The passive properties were estimated from the voltage response to hyperpolarizing current step. We estimated the resistance from the maximum amplitude of the voltage step and the capacitance by dividing the time constant by the resistance. Each group of cells showed distinct combination of resistance and capacitance (**Figure 4A**). Resistance (**Figure 4B**) was different between control and contralateral, control and ipsilateral and contra- and ipsilateral. Capacitance (**Figure 4C**) showed the same pattern of statistical differences.

Since the change in time constant could impact the firing property of the interneuron, by filtering input and intrinsic currents, we tested how the resting membrane potential and the firing threshold varied. Resting membrane potentials, around -60 mV, did not present a significant difference between control, ipsi- or contralateral tissue. We estimated the threshold current that could elicit firing by applying series of current steps and counting the number of action potentials evoked (**Figures 5A,B**). We found a significant increase in the threshold current required to elicit action potentials in contralateral tissue, where membrane time constant was the most strongly altered during epileptogenesis.

### Contribution of Interneurons to Synchronous Discharges

**FIGURE 6:**
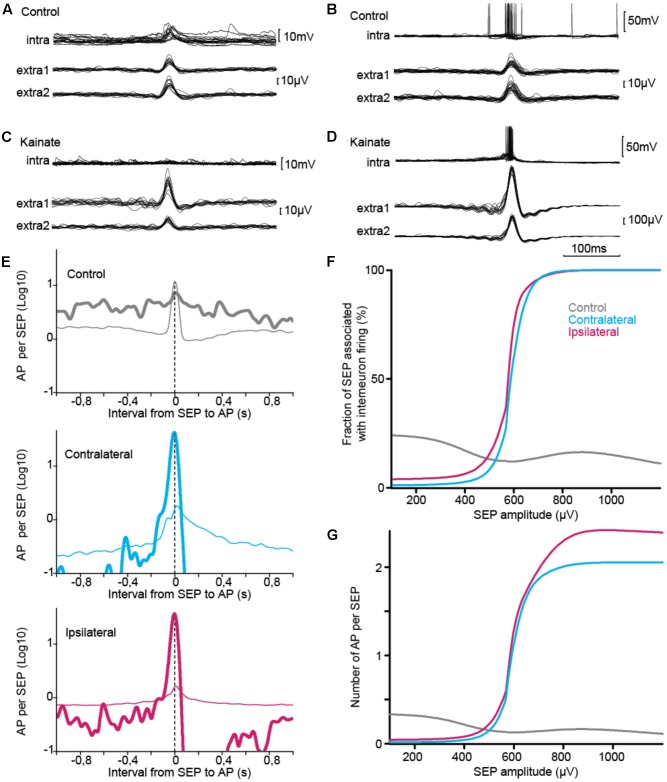
Intracellular correlate of SEP. **(A–D)** Sample traces, triggered on SEP, of interneuron membrane voltage (upper trace) and population discharges detected from a pair of extracellular electrodes (lower traces). In control, interneurons showed mixed excitatory synaptic potentials **(A)** and firing **(B)** during SEPs. **(B)** In KA-treated tissue, interneurons showed little response for SEPs of amplitude similar to those found in control tissue **(C)**, but were co-activated with the largest synchronous events **(D)**, **(A,B)** from same cell, **(C,D)** from same ipsilateral cell. **(E–G)** Interneuron firing relative to SEP, statistics over the three groups. **(E)** Interneuron firing relative to SEPs of moderate amplitude, which are common to control and KA groups (30–300 μV, thin line), and relative to SEPs of amplitude showing difference between control and KA groups (400–1200 μV, thick line). Activity normalized per length of recording in seconds. Time-locking was associated with partial synchrony in control tissue and was stronger for epileptiform discharges in KA treated tissue. **(F,G)** Firing response and number of action potentials varied in opposite directions relative to SEP amplitude in control and epileptic tissue. Similar patterns were found for ipsilateral and contralateral tissue **(E–G)**. Total number of SEP: *n*_CTRL_ = 12198, *n*_CONTRA_ = 14524, *n*_IPSI_ = 83386. Total number of action potential: *n*_CTRL_ = 4020, *n*_CONTRA_ = 231, *n_IPSI_* = 3572.

Although changes in cellular properties varied in the direction of decreased excitability in epileptic tissue, cellular contribution to population activity also depends on the numbers of cells and their connectivity. In order to address directly the question of the contribution of interneurons to synchronous discharge, we combined intracellular recording with extracellular monitoring of population discharges (**Figure 6**). In control tissue, SEPs occured mostly in association with either subthreshold excitatory synaptic potential (**Figure 6A**) or action potentials (**Figure 6B**) in the intracellular recordings. In contrast, the typical behavior of interneurons in epileptic tissue was to exhibit little synaptic potential or firing associated with SEPs of equivalent amplitude (**Figures 6C,E**). However, these neurons strongly fired when SEPs of larger amplitude were generated in the tissue. These findings suggest that interneurons in KA treated mice do not lack synaptic input, yet they are functionally tuned to selectively respond to larger population discharges.

To study the tuning of interneurons to the largest population discharges, we measured across our recordings how an interneuron responded to SEP in two distinct ranges of amplitude, 30–300 μV and 400–1200 μV (**Figure 6E**). Distributions were significantly different across groups of tissue and amplitude ranges (**Table 6**). In control tissue interneuron firing was time-locked to small SEPs, and was stronger during large SEPs but not time-locked to them. On the contrary, in epileptic tissue time-locking of interneurons was strong for large SEPs, with less firing than during small SEPs at intervals larger than 0.1–0.2 s, with a subsequent refractory period. In order to confirm this pattern, we measured, for SEP event of a given amplitude, the occurrence of intracellular firing in a time window of ±0.1 s (**Figure 6F**), and we counted the number of action potentials (**Figure 6G**). Control and epileptic tissue showed very distinct behavior of interneurons. In control tissue, interneuron firing was relatively stable over the range of amplitudes, with a decay from around 1/4 chance of firing and 0.4 action potential per SEP below 300 μV down to 1/5 and 0.2 for large SEPs. On the contrary, in epileptic tissue firing probability started below 1/10 probability with less than 0.1 action potential per SEP and increased up to one-to-one firing of around 2 action potentials per SEP above 600–700 μV. Thus, epileptic tissue interneurons appear to be irresponsive to synchrony of sparse populations but contribute to all the largest population discharges.

**Table 6:** Interneuron firing relative to SEP.

A	B	nA	nB	Maximum distance	*p*
CONTRA small	CTRL small	98055	23692	0,17	<0.01
CONTRA small	IPSI small	130833	23692	0,13	<0.01
CONTRA small	CONTRA large	593	23692	0,55	<0.01
CONTRA small	CTRL large	1209	23692	0,21	<0.01
CONTRA small	IPSI large	714	23692	0,46	<0.01
CTRL small	IPSI small	130833	98055	0,10	<0.01
CTRL small	CONTRA large	593	98055	0,40	<0.01
CTRL small	CTRL large	1209	98055	0,08	<0.01
CTRL small	IPSI large	714	98055	0,30	<0.01
IPSI small	CONTRA large	593	130833	0,48	<0.01
IPSI small	CTRL large	1209	130833	0,09	<0.01
IPSI small	IPSI large	714	130833	0,38	<0.01
CONTRA large	CTRL large	1209	593	0,46	<0.01
CONTRA large	IPSI large	714	593	0,15	<0.01
CTRL large	IPSI large	714	1209	0,31	<0.01


*Kolmogorov–Smirnov test for **Figure 6E**. Bonferroni-corrected *p*-values. nA, nB number of event for column A, B.*

## Discussion

The kainate microlesion rodent model of temporal lobe epilepsy shows a broad range of anatomical tissue remodeling (Lévesque and Avoli, 2013). In this study, tissue resected 2 weeks after the lesion showed distinct spontaneous extracellular potentials of amplitude above half a millivolt. Consistently with earlier studies, interneuron loss was found. In addition, cell body swelling was observed in CA3. In *Stratum Pyramidale* interneurons had increased capacitance and reduced resistance. Despite interneuron diversity and bilateral heterogeneity, the global effect on the sampled population was an increase in membrane capacitance, decrease in resistance, and contralateral increase in input current required to trigger action potentials. By measuring membrane potential during SEPs we found that during epileptogenesis interneurons fire little with population discharges of the same amplitude as those observed in control tissue. On the contrary, interneurons fire with each large amplitude discharge that form a signature of epileptic tissue. While the procedure revealed altered contribution of interneurons to microcircuit activity, it also has the potential to explore how their unitary field potential may contribute to the epileptic EEG. For instance, laminar and time-frequency change of SEP could be addressed with sets of basal-apical electrodes (Foffani et al., 2007). Techniques separating excitatory and inhibitory contributions could show how their balance impacts rhythmogenesis (Valero et al., 2017).

Rodent models of epileptogenesis based on kainate presents a number of variants, depending on the route of administration (intraperitoneal or focal), animal (mouse or rat), pattern of injection (single or repeated), and animal state at time of injection (awake or anesthetized, including variation with distinct anesthetics) (Tse et al., 2014; Bar-Klein et al., 2016; Twele et al., 2016; Bielefeld et al., 2017; Klee et al., 2017). In the present study mice were anesthetized with isoflurane during focal injection. Such procedure had some protective effect on rats (Bar-Klein et al., 2016). Beyond known differences between rat and mouse in kainate models (Klee et al., 2017), our injection procedure was shorter (30–40 min vs. 2 h), thus decreasing exposure to potentially protective isoflurane. In principle, the present *in vivo*–*in vitro* approach could be applied to other mouse variants of epilepsy models.

Unilateral kainate lesion of the dorsal hippocampus triggers mirror foci, on both ipsi- and contralateral sides (Sobayo and Mogul, 2013). Here, bilateral similarities and differences were found. SEPs amplitude distributions showed increased probability of larger events on both sides. In *Stratum Pyramidale*, changes in cell density and soma size were symmetric. While passive membrane properties changed bilaterally, changes were stronger on the contralateral side. This may account for the change in threshold only on the contralateral side. Interneuron co-activation with population showed bilaterally an increase of activity with SEPs amplitude. Global contribution of interneurons to network activity results from cellular densities, intrinsic properties and connectivity. While commissural fibers may account for the bilateral changes in epileptogenesis (Sobayo and Mogul, 2013), recurrent unilateral connections may be differentially altered on either side relative to the lesion.

Hippocampal interneuron diversity is a field of study in itself, as well as global tissue changes induced by a kainate lesion. Our aim was to provide an *in vivo*–*in vitro* approach to address them. Thus, we had to set aside the question of interneuron diversity in order to make the problem of interneuron functional circuit remodeling tractable experimentally, at least as a first step presented here. Our strategy was to pool all interneurons irrespective of their molecular markers, while focusing on interneurons with somas in *Stratum Pyramidale*, to constrain diversity within our cellular groups. Such diversity could potentially have masked epileptogenesis changes, as distinct interneuron types could have opposite evolutions. This strategy probably helped us uncover changes, while the variability in cellular properties could only decrease by segregating interneurons of distinct types, for example with other strains of GFP-labeled mice. Another source of variability, both at the cellular and whole-tissue levels, lies in the kainate model itself, where the lesion does not always result in the same effect. Refinements in the protocol could lower this variability (Rattka et al., 2012; Tse et al., 2014), and thus provide a more favorable context to explore the reaction of each class of interneuron, in a quantitative manner.

Changes in hippocampal GABAergic interneuronal circuits are known to play a central role in TLE epileptogenesis (Fritschy et al., 1999; Magloczky, 2010), with most of the evidence coming from histological work in both human and animal models. In animal models, injecting kainate in awake rather than anesthetized animals could reduce tissue variability and thus give sharper images of these complex transformations over time (Rattka et al., 2012). Yet, this study provides a snapshot of functional reaction of tissue to lesion, while epileptogenesis continues to develop over several months (Le Duigou et al., 2008; Shetty et al., 2009; Karunakaran et al., 2012). Enlargement of interneuron soma similar to our observation has been described in the *Dentate Gyrus* in the pilocarpine model (Halabisky et al., 2010). While we observed more interneuron loss and larger body size on the ipsilateral side, we found larger capacitance increase on the contralateral side, where threshold current increase was significant. These combined observations suggest that part of capacitance increase is generated through larger dendritic compartment.

Dentate granular cell sprouting and CA1 sclerosis form the most visible anatomical markers of TLE in the kainate model, yet CA3 lesion-induced plasticity may play a central role in sustaining pathological activities. High-resolution MRI data (Hsu et al., 2007) has shown that the first changes are observed in CA3, corresponding to focal oedema and cell death (Bouilleret et al., 2000), in agreement with the observation of kainate receptors post-synaptic to mossy-fiber contacts (Pinheiro et al., 2013). At the functional level, the CA3 recurrent network may have a triggering role in hypersynchronous activities, especially from CA3a hyperexcitable pyramidal cells (Cohen et al., 2006; Wittner and Miles, 2007). A variety of further potential mechanisms of changes in excitability associated with GABA signaling has recently been reviewed (de Curtis and Avoli, 2016; Lévesque et al., 2017), including through the indirect elevation of extracellular potassium. Strengthened population discharges in CA3 affect both recurrent connections, and projection areas. Indeed, epileptogenic lesion triggers CA3 sprouting to widespread regions of the hippocampus and the entorhinal cortex (Siddiqui and Joseph, 2005; Zhang et al., 2012). *In vivo*, these connections will propagate pathological population discharges from the hippocampus to cortical fields.

Perisomatic inhibition of principal cells by basket cells may contribute to synchronize network activity, although different mechanisms have been proposed to explain this phenomenon. In the intra-peritoneal pilocarpine model of epilepsy (Zhang et al., 2009; Thind et al., 2010) basket cells receive less excitatory synaptic drive, their pools of readily releasable vesicles are smaller, and transmission failure at basket cell-to-granule cell synapses is increased. Inhibition failure was suggested to allow runaway excitation. Another mechanism has been suggested in an acute slice model, where perisomatic inhibitory input was found to be massively involved during burst discharges, implying a simultaneous silent refractory period among principal cells and as a consequence a synchronization of their rebound discharge (Marchionni and Maccaferri, 2009). Our data presents stronger time-locking of action potential to the largest spontaneous population discharges, with a subsequent refractory period. A consensus seems to emerge as this pattern of interneuron activity with interictal discharges was recently reported in the pilocarpine model *in vivo* (Muldoon et al., 2015).

During epileptogenesis, biased coupling between interneurons and population activity may impair hippocampal rhythmogenesis (Dugladze et al., 2007), thus impacting learning and memory (Stubley-Weatherly et al., 1996). Since interneurons provide the timing framework for network rhythms, bias in their firing toward larger SEPs could hinder principal cell selectivity. Indeed, change in action potential timing selectivity in hippocampal principal cells has recently been shown to contribute to the alteration of physiological synchronous events such as Sharp-Waves Ripples and the pathological emergence of Fast Ripples (Valero et al., 2017). Furthermore, the cycle of cell loss, reactive hypertrophy and biased coupling provides a runaway path toward epilepsy which should be addressed in future therapeutic strategies, possibly with other GFP-labeled strains of mice subjected to kainate lesion.

## Author Contributions

Experiments were designed by L-AS and IC. L-AS performed electrophysiology recordings and morphology observations. IC programmed analysis software. EE produced morphological imaging. L-AS, AB, and IC analyzed the data and wrote the manuscript. All authors approved the final version of the manuscript.

## Conflict of Interest Statement

The authors declare that the research was conducted in the absence of any commercial or financial relationships that could be construed as a potential conflict of interest. The reviewer JE and handling Editor declared their shared affiliation, and the handling Editor states that the process nevertheless met the standards of a fair and objective review.
