# Supplementary material for: Disrupted Co-activation of Interneurons and Hippocampal Network after Focal Kainate Lesion
Source: Front Neural Circuits. 2017 Nov 13;11:87. doi: 10.3389/fncir.2017.00087 (PMC5693904; doi:10.3389/fncir.2017.00087)
Supplement: Supplementary file 1 [file Data_Sheet_1.docx]

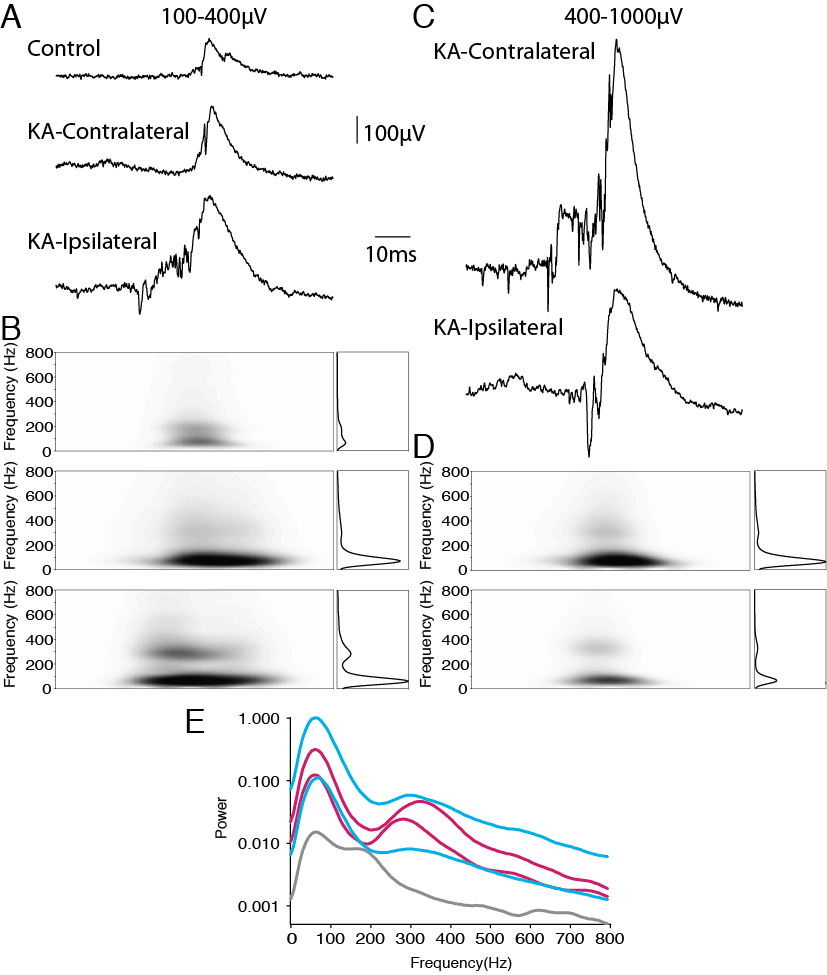


**Supplementary Figure 1 – Time-frequency signature of SEP.** A, B Example raw traces for small amplitude SEP (A) and their time-frequency spectrograms (B). C, D Example raw traces for large amplitude SEP (C) and their time-frequency spectrograms (D). Spectrogram side panels (B, D) show the global power spectrum for each time-frequency spectrogram, on a linear scale, and are normalized to maximum power. E, superimposition of the spectra in log scale, normalized to global maximum power. Spectra (B, D, E) were computed by averaging STFTs of individual SEPs as in Foffani et al. 2007, with additional high-pass filtering at 50Hz (Bessel 4th order in forward and reverse time directions) to attenuate the dominant low-frequency component and better reveal ripple or potential fast-ripple components. In our protocole frequency spread of the low frequency component overlaped with potential ripple frequency (around 200Hz), and would thus make further inference rely on modelling of the spectrum decomposition. A single peak around 300Hz in the ipsilateral group fell below the range of fast-ripple (above 400Hz).
